# Positive Psychology in Poland Between 2001 and 2020: A Review of Available Articles

**DOI:** 10.3389/fpsyg.2021.659337

**Published:** 2021-11-15

**Authors:** Marzanna Farnicka, Ludwika Wojciechowska, Łukasz Wojciechowski

**Affiliations:** ^1^Department of Social Sciences, Institute of Psychology, University of Zielona Góra, Zielona Góra, Poland; ^2^Institute of Psychology, University of Zielona Góra, Zielona Góra, Poland

**Keywords:** positive psychology, polish psychology, study review, three PP pillars, PERMA, well-being

## Abstract

The paper presents studies related to positive psychology that have been conducted in Poland. The article is based on a review of texts available in the databases of Google Scholar and EBSCO published by Polish researchers in the years 2001–2021. The selection criterion was the presence of some concepts in the title and/or abstract of articles that are constructs of positive psychology. Thirty intentionally selected articles were analyzed in terms of research objectives, variables, and measurement tools they described. In this way, a picture of research interests and methods of research on human and organization from the perspective of positive psychology was obtained. It seems that the main subjects of interest of Polish researchers were problems related to positive human qualities, life satisfaction, and mental well-being with a growing interest in positive society. The most common variable to study was life satisfaction, and the most frequently used measuring tool was the SWLS. It was concluded that there is a need for scientific exchange and integration within Polish researchers and the international community of positive psychologists.

## Introduction

The emergence of a new field of psychological research, described as positive psychology, at the end of the 20th century resulted in a discussion on the concept of good life, the importance of satisfaction and happiness for the functioning of individuals and society, and the prevention of disorders (for example: Ryan and Deci, 2001; Keyes and Waterman, 2003; Seligman, 2003; Ryff and Singer, 2004; Haidt, 2007). Polish researchers, including Czapiński (1992), Sęk et al. (1992), and Trzebińska (2008), also joined the discussion and research into positive psychology. Gradually, a group of researchers interested in positive psychology grew and more and more publications related to it appeared. A need arose to create a journal on positive psychology. Its first issue was published in 2002. It was titled “Psychology of Quality of Life” and was edited by Trzebińska (2008). An important event was also the establishment of the Polish Society of Positive Psychology^1^ in 2012, whose aim was to promote the accumulation and systematization of knowledge on high quality of life and its conditions and to scientifically develop the principles of psychological practice.

Thus, it was worth examining the current state of Polish research related to positive psychology. The authors of this article attempted to analyze the publications of Polish researchers that were written between 2001 and 2020 that is the period when Polish psychologists began to notice the importance of positive attitude (Trzebińska and Łuszczyńska, 2002). The authors decided to focus on the contribution of Polish psychologists and therefore decided to exclude monographs and review articles from the analyses. In addition, the authors’ intention was to select publications that could have a real impact on the state of knowledge about positive psychology in Poland, and for this reason, they only selected texts which were accessible for free. To create a database of articles on positive psychology, reference was made to the concepts contained in the three pillars of positive psychology, such as “mental health,” “mental well-being good life,” “quality of life,” “life satisfaction,” and “meaning in life.” Only texts containing these concepts in the title or abstract and the term “positive psychology” were selected for further analysis.

It was also assumed that in order to show the attitude of Polish researchers to positive psychology, the analysis of the texts would be determined by three aspects of the research process – research goals, variables, and measurement tools.

### Basic Assumptions of Positive Psychology

Within the assumptions of the Polish Society of Positive Psychology^2^ formulated by Polish psychologists, a synthetic understanding of positive psychology can be found. It is understood as a theoretical and empirical trend, in which the attention of researchers is focused on the abilities and skills that favor taking up challenges, developing individual dispositions, achieving a high quality of life, and coping with difficult situations. It is supposed to see an individual as a creative and productive being, enjoying life and coping with adversities (Trzebińska, 2008).

Three research pillars of positive psychology are to help to implement the above tasks (Seligman, 2003). The first pillar refers to research into positive, subjective experiencing an individual’s life in the past, present, and future. Experiencing the past positively can be described through contentment, satisfaction, and well-being, while experiencing the present can be described using the following terms: happiness (Czapiński, 1992; Ryan and Deci, 2001; Argyle, 2004; Seligman, 2005), flow (Csikszentmihalyi, 2005), flourishing (Seligman, 2005), sensual pleasures (Seligman, 2005), and positive affects (Fredrickson, 2002). Finally, the positive experience of the future can be reflected in terms of optimism and hope (Seligman, 2003).

The second pillar of positive psychology (Seligman, 2003) refers to the research of the so-called good personality, that is, a system of timeless and cross-cultural virtues, which include wisdom, humanity, courage, restraint, justice, and transcendence (Peterson and Seligman, 2004). The pillar also includes studies on strong traits (e.g., curiosity, prudence, and forgiveness) that condition the formation of virtues (*ibid.*).

The third pillar of positive psychology involves an analysis of positive society. In this regard, researchers identify institutions, such as the democratic system in the state and a properly functioning family, that allow individuals to develop positively. As Seligman (2003) emphasizes, social institutions and processes taking place in them support the abilities of individuals (e.g., commitment and responsibility) and thus create conditions for the emergence of positive emotions, which are an important capital in coping with difficult situations.

### Concepts of “Good Life”

When discussing good life, two ways of evaluating the course of life have been adopted which refer to the ancient hedonistic and eudaimonistic philosophical systems. From the hedonistic point of view, individuals experience good life when they feel pleasure and satisfaction (Czapiński, 1992; Diener et al., 2004; Trzebińska, 2008). According to the eudaimonistic approach, good life is achieved when a person shows certain valuable dispositions and aims to develop his/her potential, and his/her life is consistent with the real self (Ryff, 1989; Ryan and Deci, 2001).

Attempts to integrate both approaches were made by Keyes and Waterman (2003) who created a three-dimensional concept of well-being. Their concept took into account emotional well-being (hedonistic approach), psychological well-being developed by Ryff (1989) (eudaimonistic approach), and social well-being developed by Keyes (2003) (eudaimonistic approach) (Keyes and Shapiro, 2004). Another concept that integrates the hedonistic and eudaimonistic approaches was constructed by Seligman (2003) and called PERMA. It takes into account five dimensions of well-being (Seligman, 2011). They include “positive emotions,” “engagement,” “relationships,” “meaning,” and “accomplishment/achievement.”

Seligman (2005, p.18) emphasizes that none of the components can independently define well-being. Seligman (2005, p. 18) postulates that each component of PERMA contributes to well-being, it is pursued for its own sake, and it is defined and measured independently from the other components. Seligman (2005, p. 18) believes that only by fostering each of the PERMA elements can we work toward a true sense of well-being.

## Materials and Methods

### Article Selection Procedure

The study was based on a review of the texts available in Google Scholar and EBSCO databases which were published by Polish researchers in the years 2001–2020.

In line with the objectives of the study, the following criteria for selecting publications for the analysis were formulated:

1. The text was written by a Polish researcher.
2. The text was published between 2001 and 2020.
3. The text is an article.
4. Free (free of charge) access to the full text of the article.
5. The title and/or abstract contains the expression: “positive psychology” and at least one of the following concepts that are constructs of positive psychology: “mental health,” “mental well-being/good life,” “quality of life,” “satisfaction/life satisfaction,” and “the meaning of life.”
6. The article is of empirical nature (presentation of the results of own research). Thus, review articles were neglected.

Using the above-mentioned criteria, a target sample of 30 articles was selected for analysis.

### Research Procedure

The research procedure referred to:

- Research goals specified in the articles.
- Variables taken into account in the articles.
- Research tools used in the study.

In the first step of the analysis, the authors assigned each article to one of the three pillars described in concept of Seligman (2003). Then, each of the articles was subjected to a qualitative analysis in terms of variables and tools used in the described research.

## Publication Analysis Results

### Characteristics of Research Goals

The research goals included in the selected articles were analyzed in terms of their content and the historical aspect. The review of the research goals led to the conclusion that most of them (50%) concerned the issues of the second pillar, that is, positive human qualities and cross-cultural virtues and talents. About 30% of the goals referred to the issues of the first pillar, that is, the description of positive past or present human experiences, using the following concepts: satisfaction with various spheres of life, life satisfaction, mental well-being, and mental health. The rest of the articles (20%) contained goals referring to the third pillar, that is, positive society. Statistical verification (*p*=0.122 *Chi*^2^=4,2) does not show significant differences in the frequency of the content of the explored research goals related to the three pillars of Seligman’s concept.

The historical approach to the research goals in the analyzed articles made it possible to describe the dynamics of changes in the research interests of Polish researchers. Two periods were distinguished: from 2001 to 2011 and from 2012 to 2021. Having compared him research goals undertaken in these two periods, and some interesting differences were discovered. In the earlier period, no publications on issues related to the third pillar were found, while in the later period, an even distribution of research goals between the three pillars could be observed. There was also an increase in the number of publications in the later period (*p*=0.01 *Chi*^2^=3,6). This may indicate the growing interest of researchers in positive psychology.

### Variables

The analysis of the studied variables took into account their content and their status in the research projects presented in the articles.

Having analyzed the content of the variables, it was found that the articles most often referred to the variables that concerned the emotional aspect of human functioning (the emotional dimension of well-being, Keyes and Waterman, 2003) and the first pillar of positive psychology (Seligman, 2003), which is about positive experiences and the individual’s experiences in his/her life.

The most frequently studied variable (33%) was “general level of life satisfaction,” then “well-being” (23%) and “satisfaction in various areas of life,” that is, sex, marriage, work, and leisure time (20%). In the studied projects, these constructs most often played the role of dependent variables.

Other variables in the articles were related to concept of character strengths/positive traits of Peterson and Seligman (2004). They were as follows: “self-efficacy” (in 12% of the articles), “hope, meaning and will to live” (in 10%), and “coping with stress and resilience” (in 10%). These variables (virtues, positive traits) played the role of explanatory/independent variables. The studies most often examined the relationships between these variables and various dimensions of well-being (77%). In the remaining analyzed articles (23%), the assumptions of positive psychology were treated as a supplementary theory. As a result, despite the use of PERMA model concepts or the three-factor concept of well-being, these studies did not refer in the discussion or the conclusions to the overall models of positive psychology.

### Tools

In the analyzed publications, the most frequently used tool (36%) was the SWLS by Diener et al. (1985) in the Polish adaptation by Juczyński (2001), and it was treated as a measure of mental well-being. It should be noted that incidentally other adapted tools were also used: Meaning in Life Questionnaire (MLQ) (Steger et al., 2006), Cantril Ladder (Levin and Currie, 2014), the Scales of Psychological Well-being of Ryff (Ryff and Singer, 2004), Oxford Happiness Inventory, Minnesota Satisfaction Questionnaire, and PANAS-X (Watson and Clark, 1994).

Own research tools were used in 20% of the papers.

When comparing the research tools used in the two periods, it can be noticed that the earlier period the adaptations of research tools were less frequent. In the later period, there was a noticeable increase in the number of adapted tools (18). There is an emerging tendency to use already recognized and proven tools to a greater extent than to create new ones. This proves the involvement of Polish positive psychology researchers in global research cooperation that allows for the comparison of the obtained results and their integration. Although, the above change was not statistically significant, but it was close to the level of significance (*p*=0.101, *Chi*^2^=0.75).

## Discussion

The aim of the article was to present an analysis of the publications written by Polish researchers. The analyzed open-access articles referred to positive psychology and were available in the databases of scientific articles.

The analysis of the research goals revealed that over the period of 20years, researchers were interested in all three pillars of positive psychology. Based on the historical analysis, it can be indicated that along with the development of positive psychology in Poland, areas within the third (social) pillar were explored more and more often. Studies conducted in the earlier period (2001–2011) focused mainly on the first and second pillar. In the later period (2012–2021), one could notice an increase in the number of articles, the diversity of their topics, an expansion of the research issues, and the content of the research goals.

As far as variables are concerned, it was found that the main concepts present in the articles were well-being and life satisfaction. The majority of the studies focused on variables related to emotional well-being (happiness, overall life satisfaction, or satisfaction in various areas of life). It was also noticed that this aspect of well-being was often equated with the broad concept of mental well-being. This tendency is noticeable both in Poland and in the world (Czapiński, 1992; Fredrickson, 2002; Diener et al., 2004; Haidt, 2007; Ruggeri et al., 2020).

Also, the assumptions of the positive psychology concept were used in the analyzed articles in different ways. On the one hand, the articles contained extensive, coherent research models using the existing theory or showing new possibilities of this approach (e.g., Wojciechowska, 2007 – discussion on the concept of psychological well-being and suggesting the concept of personality well-being). On the other hand, the categories of positive psychology were treated as a collection of useful and fashionable concepts, and the concepts of positive development were used to include this thread in research conducted within a different approach. As a result, despite the use of PERMA model concepts or the three-factor concept of well-being, some articles did not refer in the discussion or conclusions to the overall models of positive psychology.

It seems that the described phenomenon may be a consequence of the low level of awareness of the possibilities and conceptualization offered by positive psychology. One can also conclude that there is certain conservatism in created research plans, and only a small group of experienced researchers apply research plans fully anchored in positive psychology models. Therefore, on the basis of the analysis of available publications, it is possible to predict the next stage in the development of positive psychology in Poland: There will be comprehensive research plans based on positive psychology, which will meet Seligman (2003) postulate and will cover all dimensions of psychological well-being, all PERMA elements, and the concept of character virtues and strengths by Peterson and Seligman (2004).

The analysis of the research tools showed that the SWLS was the most frequently used tool. This may result from the popularity of this tool related to its availability, simplicity of testing, and short time needed to obtain results.

Based on the historical analysis, it can be noted that there is a growing tendency to use other, already recognized and proven tools. This facilitates the involvement of Polish positive psychology researchers in global collaboration, and this makes the result comparison possible. On the other hand, it is worth emphasizing the fact that efforts are made to create new tools in the eudaimonistic trend (Ryff, 1989; Wojciechowska, 2007; Karaś et al., 2013; Krok, 2013).

Summarizing, it can be stated that although the authors of this study had the limited material from open-access databases, they were able to outline the research interests of Polish scientists dealing with positive psychology between 2001 and 2021. The discovered interests of Polish researchers indicate that Polish positive psychologists are active and creative in positive psychology as a psychological metatheory. Moreover, it was also possible to present/define specific trends and development paths of positive psychology in Poland (along with the most popular theories or measurement tools).

### Limitation and Conclusion

This text was prepared on the basis of 30 selected articles presenting empirical research. The selected articles were available in two open-access databases. Before starting the selection procedure, it was expected to find at least 100 articles meeting the adopted criteria. However, only 30 articles met them. The selection method excluded non-open-access articles. We are aware of the existence of other databases with a greater number of publications related to positive psychology, but access to these databases is not free of charge.

On the basis of the presented review of publications, it can also be concluded that Polish scientists do not control whether their publications are available to a wide audience. The scarce dissemination of the scientific achievements of Polish researchers makes the contribution of Polish science to the development of positive psychology almost unknown. Therefore, this problem requires greater care and efforts leading to a greater internationalization of the scientific achievements of Polish positive psychologists.

The intensification of research into positive psychology would contribute to the study of the conditions and processes necessary for the maximum functioning and development of individuals, groups, and institutions. This would enable the creation of consortia with experienced researchers who use extensive research plans. As a result, the integration of results from micro-areas related to specific age or problem groups into knowledge useful for positive psychology would be possible.

## Author Contributions

All authors listed have made a substantial, direct and intellectual contribution to the work, and approved it for publication.

## Conflict of Interest

The authors declare that the research was conducted in the absence of any commercial or financial relationships that could be construed as a potential conflict of interest.

## Publisher’s Note

All claims expressed in this article are solely those of the authors and do not necessarily represent those of their affiliated organizations, or those of the publisher, the editors and the reviewers. Any product that may be evaluated in this article, or claim that may be made by its manufacturer, is not guaranteed or endorsed by the publisher.

## Supplementary Material

The Supplementary Material for this article can be found online at: https://www.frontiersin.org/articles/10.3389/fpsyg.2021.659337/full#supplementary-material

## Footnotes

^1^ https://ptpp.org.pl/

^2^ https://ptpp.org.pl/
