# Supplementary material for: Positive Psychology in Poland Between 2001 and 2020: A Review of Available Articles
Source: Front Psychol. 2021 Nov 15;12:659337. doi: 10.3389/fpsyg.2021.659337 (PMC8996460; doi:10.3389/fpsyg.2021.659337)
Supplement: Supplementary file 1 [file Data_Sheet_1.docx]

Appendix 1. Material specification - articles obtained in the results of the selection procedure

1. Chanduszko-Salska, ]., Chodkiewicz, J. (2010). Zadowolenie z życia a poczucie własnej skuteczności, wsparcie społeczne oraz stan zdrowia u kobiet z nadwagą i otyłością. Endokrynologia. Otyłość i Zaburzenia Przemiany Materii, 6 (4),p. 171-178.

2. Chudnicki, A. (2021). Dobrostan psychiczny i poczucie sensu życia a wypalenie zawodowe funkcjonariuszy Służby Więziennej. Badania wstępne. *Annales Universitatis Mariae Curie-Skłodowska, sectio J – Paedagogia-Psychologia, 33*(4), 209-221. doi:<http://dx.doi.org/10.17951/j.2020.33.4.209-221>

3. Chudzicka-Czupła, A., & Zalewska- łunkiewicz, K. (2018). Wybrane korelaty dobrostanu psychicznego kandydatów na asystentów zdrowienia, *Czasopismo Psychologiczne,* 24(3), 573-583. DOI: 10.14691/CPPJ.24.3.573

4. Czarnecka-Iwańczuk M., , Stanisławska-Kubiak M., Mojs E., Wilczak M., Samborski W. (2012). Objawy menopauzy a satysfakcja z życia i samoocena wśród kobiet, *Przegląd Menopauzalny*, 6 (6), 468-473. DOI:  [10.5114/pm.2012.32539](http://dx.doi.org/10.5114/pm.2012.32539)

5. Drogosz, M., Dziuba, M., Prażmowska, M. (2006). Styl autonarracji a wybrane aspekty funkcjonowania członków różnych grup społecznych. Psychologia Jakości Życia, 5, 213–236.

6. Jakubowska, E., Jakubowski K., Cipora E.  (2010).  Satysfakcja z życia chorych z cukrzycą, *Problemy Higieny i Epidemiologii*,  91 (2), pp. 8-313.   http://www.phie.pl/phe.php?opc=AR&lng=pl&art=443

 7. Kanadys, K., Rogowska, J., Lewicka, M., Wiktor, H. (2015). Satisfaction with life among pregnant women. *Medycyna  Ogólna i  Nauki o  Zdrowiu*, 21(1), 45-48. <https://doi.org/10.5604/20834543.1142358>

8. Karaś, D. , Kłym,M., Cieciuch, J. (2013). Eudajmonistyczny dobrostan psychiczny a kształtowanie poczucia tożsamości w sferze edukacyjnej i zawodowej. *Psychologia Rozwojowa*, 18(1), 87-10.

9. Kaźmierczak M., Pastwa-Wojciechowska B., Błażek M. (2013). A multidimensional model of empathy, and the occurrence of personality disorders and stress in social settings. *Acta Neuropsychologica,* 11(2), 113-125.

 10. Kosiba, G., Gacek, M., Bogacz-Walancik, A., Wojtowicz, A. (2017). Zachowania prozdrowotne a satysfakcja z życia studentów kierunków nauczycielskich. *Teraźniejszość – człowiek – eduka*cja, 20, 2(78), 79-93.

11. Kossakowska, M., Kwiatek, P., Stefeniak, T. (2013). Sens w życiu. Polska wersja kwestionariusza MLQ. *Psychologia Jakości Życia*,  2013; 2, 111-115.

12. Krok D. (2013). Nadzieja jako predyktor wymiarów dobrostanu psychicznego. *Polskie Forum Psychologiczne*, 18 (2), 157-172.

13. Łaguna, M. (2012). Satysfakcja z życia i satysfakcja z pracy a motywacja do podejmowania szkoleń: Doniesienie z badań, *Psychologia Jakości Życia*,  12( 2), 163–172.

14. Łukasik, I., & Witek, A. (2018). Nastawienie na sukces w kontekście pozytywnej orientacji i poczucia własnej skuteczności. *Annales Universitatis Mariae Curie-Skłodowska, sectio J – Paedagogia-Psychologia, 31*(2), 301-312. doi:<http://dx.doi.org/10.17951/j.2018.31.2.301-312>

15. Malina, A. (2011). Styl przywiązania młodych kobiet a ich satysfakcja z życia w różnych fazach rozwoju rodziny. *Psychologia Rozwojowa*, 16(1), 41-55. doi:<https://doi.org/10.4467/20843879PR.11.003.0176>

16. Martowska, K., Matczak A. (2013). Pomiar kompetencji społecznych - prezentacja nowego narzędzia diagnostycznego. *Psychologia Jakości Życia*, 1, 43-56.

17. Maruszczak, M., Brygoła, E. (2019). Spostrzegane wsparcie społeczne a dobrostan psychiczny u młodzieży wychowującej się i niewychowującej się w domu dziecka. Przekonanie o zmienności cech jako możliwy mediator zależności". *Psychologia Rozwojowa*, 2019, 71-83. doi:<https://doi.org/10.4467/20843879PR.19.004.10595>

18. Mularska-Kucharek, M. (2020). Czy warto ufać innym? Wybrane funkcje zaufania społecznego na przykładzie zbiorowości miejskich. *Studia Miejskie*, *21*, 101-116. www. czasopisma.uni.opole.pl/index.php/sm/article/view/2541

19. Pajor, A. , Broniarczyk-Dyła G., Świtalska J. (2015). „Satysfakcja z życia, poczucie własnej wartości oraz ocena zdrowia psychicznego u osób z tatuażem lub piercingiem”, *Psychiatria Polska*, 49(3): 559–573. DOI: <http://dx.doi.org/10.12740/PP/27266>

20. Paszkowska-Rogacz, A. (2019). Perspektywa temporalna młodych dorosłych a ich zadowolenie z życia. Moderujący efekt poczucia dorosłości. *Psychologia Rozwojow*a, 24(3), pp.  77-95. doi:<https://doi.org/10.4467/20843879PR.19.018.11296>

21. Pietruszewski, B., & Siwy-Hudowska, A. (2013). Osobowość, podejmowanie zachowań ryzykownych a satysfakcja z życia młodych osób po przeszczepie serca. *Psychologia Jakości Życia*, *12*(2), 133–148. <http://doi.org/10.5604/16441796.1090790>

22. Poprawa, R. (2001). Zasoby osobiste w radzeniu sobie ze stresem. W: G.Dolińska-Zygmunt (red.), *Podstawy psychologii zdrowi*a [s.103-141]. Wrocław: Wydawnictwo Uniwersytetu Wrocławskiego.

23. Sikorska, I., Lipp, N., Stadtmuller, K., Mostowik, J. (2019). Szczęśliwe życie w okresie późnej dorosłości. Znaczenie motywu indywidualnego. *Psychologia Rozwojowa*, 2019, 29-42. doi:<https://doi.org/10.4467/20843879PR.19.001.10592>

24. Sobol, M., & Oleś, P. (2002). Orientacja temporalna carpe diem a poczucie satysfakcji z życia. *Przegląd Psychologiczny*, *45*(3), 331-346.

25. Terelak, J. F., & Borkowska, A. (2018). Satysfakcja z pracy jako źródło dobrostanu psychicznego u polskich emigrantów konsumpcyjnych w Kanadzie. *Studia Psychologica: Theoria Et Praxis*, (7), 101-122.  <https://czasopisma.uksw.edu.pl/index.php/sp/article/view/2652>

26. Wnuk M. (2007). Abstinence and the length of time spent in a self-help group and mental wellbeing of alcohol dependent persons The Twelve-step Program mediating role.  *Alkoholizm i Narkomania*,  20(4), 395- 416.

27. Wojciechowska, L. (2007). The theory of well-being in developmental research on the family: The well-being of parents in the empty nest stage. *Polish Psychological Bulletin*, *3*(38), 166-174.

28. Wojciechowska, L. (2016). Przyjaźń a subiektywny dobrostan psychiczny adolescentów. *Psychologiczne Zeszyty Naukowe*, (1), 75-90.

29. Wycisk, J., Warawan, L. (2013). Satysfakcja małżeńska u kobiet niepłodnych z uwzględnieniem stylu radzenia sobie ze stresem. *Psychologia Jakości Życia,* 12, 1, 75-89.

30. Żechowski, C., Cichocka, A., Rowiński, T., Mrozik, K., Kowalska-Dąbrowska, M., Czuma, I. (2018). Style przywiązania a zdrowie psychiczne osób dorosłych w populacji ogólnej - badanie pilotażowe. *Psychiatria, 15*(4), 193-198.
